# An Analytical Model for the Performance Analysis of Concurrent Transmission in IEEE 802.15.4

**DOI:** 10.3390/s140305622

**Published:** 2014-03-20

**Authors:** Cengiz Gezer, Alberto Zanella, Roberto Verdone

**Affiliations:** 1 Vestel Electronics, 45030, Manisa, Turkey; 2 Institute of Electronics, Computer and Telecommunication Engineering (IEIIT), National Research Council (CNR), 40126, Bologna, Italy; E-Mail: alberto.zanella@cnr.it; 3 Electrical, Electronic, and Information Engineering (DEI), University of Bologna, 40126, Bologna, Italy; E-Mail: roberto.verdone@unibo.it

**Keywords:** concurrent transmissions, capture effect, IEEE 802.15.4

## Abstract

Interference is a serious cause of performance degradation for IEEE802.15.4 devices. The effect of concurrent transmissions in IEEE 802.15.4 has been generally investigated by means of simulation or experimental activities. In this paper, a mathematical framework for the derivation of chip, symbol and packet error probability of a typical IEEE 802.15.4 receiver in the presence of interference is proposed. Both non-coherent and coherent demodulation schemes are considered by our model under the assumption of the absence of thermal noise. Simulation results are also added to assess the validity of the mathematical framework when the effect of thermal noise cannot be neglected. Numerical results show that the proposed analysis is in agreement with the measurement results on the literature under realistic working conditions.

## 1. Introduction

Among the possible medium access control (MAC) techniques for wireless communication systems, the simplicity of random access schemes (*i.e.*, ALOHA, carrier sense multiple access (CSMA)) make them suited to be implemented in several standards for short range applications [1,2]. Even though mitigation methods can be introduced in random access MAC (i.e., carrier sense multiple access with collision avoidance (CSMA-CA)), collisions are not completely avoidable. Nevertheless, some receivers have the ability to correctly receive a signal despite a significant level of co-channel interference, and collisions do not always lead to a total loss of the collided packets. This co-channel interference tolerance is called capture effect [3]. In the presence of concurrent transmissions at the same carrier frequency (collisions), packet capture may happen even for low values of the signal-to-interference ratio (SIR).

The first papers in the literature about the capture effect mostly consider Frequency Modulation (FM) demodulators [3–5]. Later, the capture effect has been also studied in a variety of transceivers and MAC schemes, including ALOHA networks [6–8], IEEE 802.11 devices [9–11], Bluetooth radios [12] and cellular systems [13]. Focusing on IEEE802.15.4, several papers describing experimental results can be found in the literature [14–22]. To give some examples, in [14], the packet capture probability of a Chipcom CC1000 transceiver [15] is measured with the aim of exploiting the capture effect for collision detection and recovery. Another study [16], carried out again with CC1000 transceivers, which work in the sub-1 GHz band, obtained a threshold for the capture probability for the case of one interferer, but unstable results for the multiple interferers. This early work seems to suggest that the number of interferers might have an important effect on the capture probability with CC1000 devices. However, in contrast to the previous CC1000 measurements, successive studies, carried out with Chipcon CC2420 transceivers [17], which operate in the 2.4 GHz industrial, scientific and medical (ISM) band, show that the capture effect depends on the total interfering power, but it is independent of the number of interferers [18,21]; therefore, in our mathematical analysis, we assume that the performance is independent of the number of interferers for the 2.4 GHz physical layer (PHY) of IEEE 802.15.4, and as a result, we use only one interfering signal for the mathematical evaluation. A behavior on the packet capture similar to that reported in [16,18] is also observed in [20] with Freescale MC1224 transceivers [22], which, again, operate in 2.4 GHz. The experiments conducted in interferer power-dominant (with respect to the noise) environments in [16,18–20] show a couple of common behaviors: (i) the receiver starts capturing the useful packet when signal-to-interference-plus-noise ratio (SINR) goes beyond 0 dB; (ii) packet reception rate (PRR) reaches one for values of SINR larger than 4 dB (please see in [16], Figure 5 and Figure 16, for CC1000 and CC2420, respectively; in [18], Figure 4 for CC2420; in [19], Figure 7c for CC2420; and in [20], Figure 3 for MC13192).

Although the experimental results in [16,18–20] agree with each other, there is no theoretical model, purely based on mathematical analysis, which can be applied to the 2.4 GHz PHY of IEEE 802.15.4 to explain such characteristics. Motivated by this consideration, we propose an analytical framework to investigate the behavior of the IEEE 802.15.4 2.4 GHz PHY layer. A review of low-rate wireless personal area network (LR-WPAN) solutions, including IEEE 802.15.4, can be found in [23].

The impact of interference in wireless sensor networks plays a very important role and can severely degrade the overall performance of the network and the efficiency of the upper layers. In our opinion, this aspect has not been sufficiently addressed in past years. In a dense sensor network deployment, where many nodes are periodically sending data to the sink, concurrent transmissions are highly probable. However, the probability of having a collision because of more than two concurrent transmissions is relatively low [18,20], thanks to CSMA-CA. In such conditions, the performance of the receiver depends on the overall amount of interferer signal energy and does not change with the number of interferers [18,21]. Hence, solely the impact of one interferer on the capture probability will be considered in the mathematical analysis.

On the other hand, we believe this study can also contribute to the identification of signal reception models for network simulators. In particular, as shown in Table 1, most common network simulators use signal-to-noise ratio threshold (SNRT)- or bit error rate (BER)-based models in order to decide the correct reception of a packet.

In SNRT-based models, the packet is correctly received if the signal-to-noise ratio (SNR) is larger than a given threshold, whereas, in BER-based models, the packet reception decision is based on the BER, which is determined probabilistically depending on the value of the SNR. These models are rather simple, but have some drawbacks. In particular, SNR-based models do not take into account the impact of interference. This latter effect can be considered, in principle, by BER-based models, but the impact of the waveform of the interferer signals should be carefully considered, as it plays a significant role. Typically, conventional interference models are based on the assumption that the disturbance can be modeled as a Gaussian random variable; unfortunately, this is not the case of IEEE 802.15.4 systems, where only a limited number of strong interferers is present. To counteract this problem, we mathematically analyze the impact of the waveform of the interferer on packet reception and obtain curves that are organized as specific look-up tables. Figures, such as those derived in Figures 4 and 6, can be used to provide accurate PHY models for network simulators. In that case, the conventional on-off behavior of SNRT-based models can be replaced by a probabilistic model, where the actual value of SIR leads to a given probability of packet loss. In other words, we provide a SIR-based signal reception model for the interference-dominant environments, where noise is not the serious cause of packet loss (i.e., enough transmit power is used or nodes are using the best links to reach the destinations in a dense sensor network deployment). Furthermore, Figure 7 shows that, in the case of non-coherent detection in an interferer-dominant environment, an on-off model can be also applied. In any case, behavior changes when thermal noise cannot be neglected. As a conclusion, the results of this paper on chip error rate (CER) and PRR (see Figures 6 and 7) can be used within network simulators in terms of look-up tables. That allows a fast characterization of the behavior of the PHY layer.

The computational complexity of the model for the coherent detection is O(1) (in big O notation). This makes it usable without intensive computational effort. For the non-coherent case, we show that the performance curve has a step-like behavior with the threshold at 0 dB. This simple model can capture the behavior of the non-coherent case without any computational effort.

The rest of the paper is organized as follows: Section 2 describes CSMA-CA and the 2.4 GHz PHY of the IEEE 802.15.4 Standard. Sections 3 and 4 evaluate CER for coherent and non-coherent offset quadrature phase shift keying (O-QPSK), respectively. Section 5, obtains PRR in concurrent transmission by finding an upper bound, which transforms CER to data symbol error rate (DSER).

## 2. System Description

Two different approaches are foreseen in IEEE 802.15.4 to coordinate the data traffic: beacon-enabled mode and non-beacon-enabled mode [2]. In the former, periodic beacons are sent by the coordinator to synchronize the channel access, while in the latter, no synchronization is required. CSMA-CA is used in both modes, but in a slightly different way. In the beacon-enabled mode, every node synchronizes itself to the backoff slots determined by the coordinator; for this reason it is named slotted CSMA-CA. In non-beacon-enabled mode, every node has its own backoff slot timing, so that it is called unslotted CSMA-CA. Default values of the parameters and the number of sensing phases before assessing the channel idle are different in slotted and unslotted versions as a result of synchronization (*i.e.*, two backoff slots in slotted, one backoff slot in unslotted). In both modes, each node waits for a random number of backoff slots, then channel sensing is performed. If the channel is found to be free, the node immediately starts the transmission. If the channel is found to be busy, the node turns back to the backoff state again. There is a maximum number of attempts a node can try to sense the channel. When this maximum is reached, the algorithm ends with a failure. Even though CSMA-CA avoids collisions by sensing the channel before transmitting a packet, a hidden terminal effect or sensing the channel as idle exactly at the same time by more than one node can cause a collision. As discussed in the previous Section, a collision does not always lead to a total loss. One of the packets, most probably the one with the better signal strength, can be successfully received.

IEEE 802.15.4 PHY uses direct sequence spread spectrum (DSSS), and it operates over different ISM bands: the 868 MHz (EU) ISM band, the 915 MHz (US) ISM band and the 2.4 GHz global ISM band. Because of the global availability of the higher frequency band, currently transceiver manufacturers mostly have products working in this band (*i.e.*, Chipcon CC2420, Freescale MC13224, *etc.*). In the paper, we focus on the 2.4-GHz frequency band, which utilizes half-sine shaped O-QPSK modulation. In the PHY layer of the 2.4 GHz band, the signal is first modulated by forming a data symbol of four bits, and then, this symbol is mapped to 32-chip long sequences, obtaining 16 data symbols. The data symbol set modulated at the carrier frequency, *f_c_*, and carrier phase, *ϕ_C_*, can be written as:

(1) $$\begin{array}{cc} s_{Ci}(t)=I_{i}(t)\cos(2\pi f_{c}t+\phi_{C})-Q_{i}(t)\sin(2\pi f_{c}t+\phi_{C}) & i=0,1,\ldots ,15 \end{array}$$

For each chip sequence, even-indexed chips are modulated to the in-phase (I) carrier, and odd-indexed chips are modulated to the quadrature-phase (Q) carrier of O-QPSK. The offset between the I-phase and Q-phase is formed by delaying the Q-phase symbols one chip duration with respect to the I-phase. *I*(*t*) and *Q*(*t*) for each data symbol can be written as in [25]:

(2) $$I_{i}(t)=\overset{15}{\underset{n=0}{\text{∑}}}c_{2n}^{i}h(t-2nT_{c})$$

(3) $$Q_{i}(t)=\overset{15}{\underset{n=0}{\text{∑}}}c_{2n+1}^{i}h(t-(2n+1)T_{c})$$

where $c_{k}^{i}$ is ±1 with respect to the chip value and *i* is the index of the data symbols. The function, *h*(*t*), is half-sine shaped and can be expressed as:

(4) $$h(t)=\{\begin{array}{ll} \cos\left(\frac{\pi t}{2T_{c}}\right) & \text{if}-T_{c}\leq t<T_{c}\\ 0 & \text{otherwise} \end{array}$$

Finally, the complex baseband signal is given as:

$${\tilde{s}}_{C_{i}}(t)=I_{i}(t)+jQ_{i}(t);$$

the corresponding data symbol set in time domain will be expressed by using the complex envelope as:

(5) $$s_{Ci}(t)=ℜ\left[{\tilde{s}}_{C_{i}(t)}\exp\left(j(2\pi f_{c}t+\phi_{C})\right)\right]$$

where ℜ[·] is the real part of the complex expression.

## 3. Probability of Chip Error in Coherent O-QPSK

In this section, we consider coherent communication and evaluate the chip error probability when there is one interferer with an unknown carrier phase and symbol timing. As the experiments in [16,18–20] suggest, we assume that the signal of the interferer is additive, and the probability of error is independent of the number of interferers. As a consequence, we will consider one interfering signal. Under such an assumption, the received O-QPSK modulated signal, in the time interval −*T_c_* ≤ *t* < *T_c_*, can be written as:

(6) $$\begin{array}{cc} r(t)=ℜ\left[{\tilde{s}}_{C}(t)\exp\left(j(2\pi f_{c}t+\phi_{C})\right)\right]+ℜ\left[{\tilde{s}}_{I}(t)\exp\left(j(2\pi f_{c}t+\phi_{I})\right)\right] & -T_{c}\leq t<T_{c} \end{array}$$

where *s͂_C_* (*t*) and *s͂_I_* (*t*) are the complex envelopes of the useful and interferer signals and *ϕ_C_* and *ϕ_I_* are the carrier phases of the useful and interferer signals, respectively. There is no phase difference between the useful transmitter and the receiver, since we are assuming coherent demodulation. Therefore *ϕ_C_* = 0, but the carrier phase of the interferer, *ϕ_i_*, is unknown. After some algebra, Equation (6) can be expressed as:

(7) $$\begin{array}{cc} r(t)=ℜ\left[\tilde{r}(t)\exp(j2\pi f_{c}t)\right] & -T_{c}\leq t<T_{c} \end{array}$$

where the complex envelope is:

(8) $$\begin{array}{cc} \tilde{r}(t)={\tilde{s}}_{C}(t)+{\tilde{s}}_{I}(t)\exp(j\phi_{I}) & -T_{c}\leq t<T_{c} \end{array}$$

**Figure 1.**
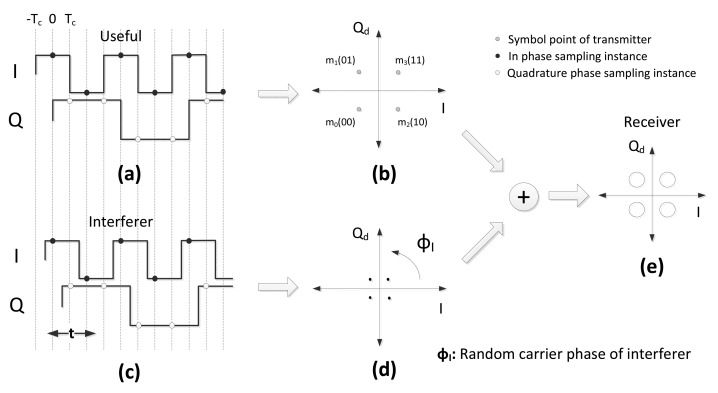
The impact of the interferer. (**a**) Useful signal; (**b**) Q*_d_*-I domain representation of useful signal; (**c**) Interferer signal; (**d**) Q*_d_*-I domain representation of interferer signal; (**e**) Q*_d_*-I domain representation of received signal.

In the case of coherent demodulation, the I and Q components should be sampled at the instance corresponding to the maximum signal amplitude; hence for a sequence of chips, the I-phase should be sampled at the instants *t_I_n__* = 2*nT_c_* (see Equation (2)) and the Q-phase at the instants *t_Q_n__* = (2*n* + 1)*T*_c_ (see Equation (3)), where *n* = 0, 1, 2,…. This results in sampling the I and Q components of the useful signal at the indicated sampling instances in Figure 1a and Figure 3a. Due to the delayed sampling between the I and Q components, we will consider a Q-I constellation plane, where the Q component is delayed by *T_c_* seconds with respect to the I component. We named the resulting constellation plane Q*_d_*-I to emphasize the delay. In the next two subsections, we derive the CER for the received signal in Equation (8), as a function of SIR, considering two cases: without and with pulse shaping. Thermal noise is neglected in the analysis, as we want to investigate solely the effect of interference on CER. The effect of noise will be considered in Section 5 to compare the analysis with the experimental results.

### 3.1. Without Pulse Shaping

When we take the case of no pulse shaping into account, the shaping function, *h*(*t*), is a non-return to zero (NRZ) rectangular pulse, whose expression is:

(9) $$h(t)=\{\begin{array}{ll} 1 & -T_{c}\leq t<T_{c}\\ 0 & \text{otherwise.} \end{array}$$

Accordingly, the complex envelope of the received signal in Equation (8) on the Q*_d_*-I constellation plane can be written as:

(10) $$\begin{array}{cc} \tilde{r}(t)=\left(\pm \sqrt{\frac{C}{2}}\pm j\sqrt{\frac{C}{2}}\right)+\left(\pm \sqrt{\frac{I}{2}}\pm j\sqrt{\frac{I}{2}}\right)h(t)\exp(j\phi_{I}) & \begin{array}{cc} -T_{c}\leq t<T_{c}, & 0\leq \phi_{I}<2\pi \end{array} \end{array}$$

where *C* and *I* represent the energies of the useful and interferer signals, respectively. ϕ*_I_* is the carrier phase of the interferer. Since the demodulation is coherent, perfect knowledge of the carrier phase and symbol timing is assumed for the useful signal. These parameters are supposed to be uniformly distributed random variables for the interfering signal. The pulse shaping function is constant throughout a chip duration; thus, random symbol timing of the interferer, *t*, does not give any contribution to the performance. On the contrary, the presence of an interferer carrier phase has a significant impact on the received signal, because *ϕ_I_* rotates the interfering signal on the Q*_d_*-I domain, as indicated in the second addend of Equation (10). The geometric representation of Equation (10) on the Q*_d_*-I constellation plane is depicted in Figure 1.

With respect to the transmitted symbol, the first addend in Equation (10) will be one of the constellation points on the Q*_d_*-I domain, as shown in Figure1b, while the second addend in Equation (10) represents the interfering signal, which can be any of the points shown in Figure 1d, but rotated according to the interferer carrier phase, *ϕ_I_*. The final sum in the equation can be interpreted as in Figure 1e; the sampling point of the received signal is located in one of the quadrants in the Q*_d_*-I plane, in agreement with the transmitted useful symbol, but, due to the presence of the interferer, it is shifted to a random location over the circle with radius $\sqrt{I}$ and the center at $\left(\pm \sqrt{\frac{C}{2}},\pm j\sqrt{\frac{C}{2}}\right)$. The amplitude of the useful signal on the Q*_d_*-I plane is $\sqrt{C}$, and the amplitude of the interferer signal is $\sqrt{I}$. In the following parts, *P_c_* and *P_s_* will denote the probability of chip error and the probability of O-QPSK symbol error, respectively If we consider an ideal maximum likelihood demodulator [26], three distinct cases need to be investigated.

#### 3.1.1. Case (a): $\sqrt{I}<\sqrt{C/2}$

**Figure 2.**
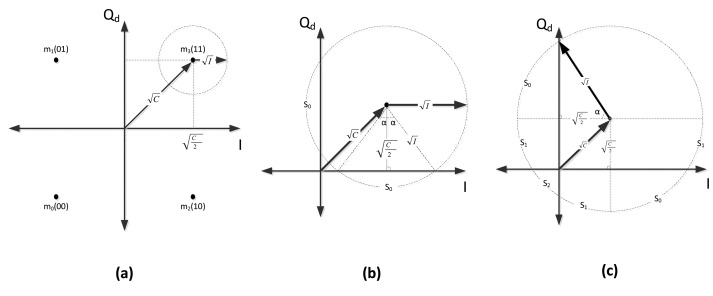
Coherent Cases. (**a**) $\sqrt{I}<\sqrt{C/2}$; (**b**) $\sqrt{C/2}\leq \sqrt{I}<\sqrt{C}$; (**c**) $\sqrt{C}\leq \sqrt{I}$.

When $\sqrt{I}$ is smaller than $\sqrt{C/2}$, as shown in Figure 2a, the received signal never crosses other symbol regions. As a consequence, the demodulator will never make an error, and therefore:

$$\begin{array}{ll} P_{c} & =0\\ P_{s} & =0 \end{array}$$

#### 3.1.2. Case (b): $\sqrt{C/2}\leq \sqrt{I}<\sqrt{C}$

When $\sqrt{I}$ is larger than (or equal to) $\sqrt{C/2}$, the demodulator will start deciding erroneously, because of the stronger signal energy of the interfering component, which forces the received signal to be sampled in one of the adjacent quadrants on the constellation plane, as shown in Figure 2b. In such a condition, the probability of symbol error will be given by:

$$P_{s}=\frac{S_{0}}{\pi }=2\frac{2\alpha }{2\pi }=\frac{2\alpha }{\pi }$$

where *S*_0_ is the arc of the circle shown in Figure 2b, the angle, *α*, in radians, is equal to *S*_0_/2, and *α* is:

(11) $$\alpha =\arctan\sqrt{\frac{I-(C/2)}{\sqrt{C/2}}}$$

The sampling point of the received signal can only pass to the adjacent quadrants, and the receiver makes a one chip error per each transmitted symbol; as a result, *P_c_* will be the half of *P_s_*:

$$P_{c}=\frac{4\alpha }{4\pi }=\frac{\alpha }{\pi }$$

#### 3.1.3. Case (c): $\sqrt{C}\leq \sqrt{I}$

For increasing values of $\sqrt{I}$, the circle will move to the other side of the origin (see Figure 2c). *α* can still be written as in Equation (11), but now:

$$\begin{array}{ll} S_{0} & =\alpha \\ S_{1} & =\pi /2-\alpha \\ S_{2} & =2\alpha -\pi /2 \end{array}$$

When the received signal is in *S*_2_, the receiver makes two chip errors per each transmitted symbol. On the other hand, when it is in *S*_0_ or in *S*_1_, the receiver makes one chip error per each transmitted symbol. Regarding the number of chip errors in the different quadrants, *P_s_* and *P_c_* can be written as:

$$\begin{array}{ll} P_{s} & =\frac{2(S_{0}+S_{1})+S_{2}}{2\pi }=\frac{\alpha }{\pi }+\frac{1}{4}\\ P_{c} & =\frac{2(S_{0}+S_{1})}{2\pi }\frac{1}{2}+\frac{S_{2}}{2\pi }=\frac{\alpha }{\pi } \end{array}$$

The computational complexity of *P_s_* and *P_c_* is *O*(1) in big O notation, since *C* and *I* are constant values.

### 3.2. Half-Sine Pulse Shaping

Using the pulse shaping given by Equation (4), the received signal in Equation (8) can be written, on the Q*_d_*-I plane, as:

(12) $$\begin{array}{cc} \tilde{r}(t)=\left(\pm \sqrt{\frac{C}{2}}\pm j\sqrt{\frac{C}{2}}\right)+\left(\pm \sqrt{\frac{I}{2}}\pm j\sqrt{\frac{I}{2}}\right)\cos\left(\frac{\pi t}{2T_{c}}+\phi_{I}\right) & \begin{array}{cc} -T_{c}\leq t<T_{c}, & 0\leq \phi_{I}<2\pi \end{array} \end{array}$$

where the first addend represents the useful symbol and the second addend represents the interferer symbol. In the variable, *t*, in Equation (12), is the sampling instance for the interferer symbol, and *ϕ_I_* is the carrier phase of the interferer, which shifts the pulse shaping function on the Q*_d_*-I domain.

**Figure 3.**
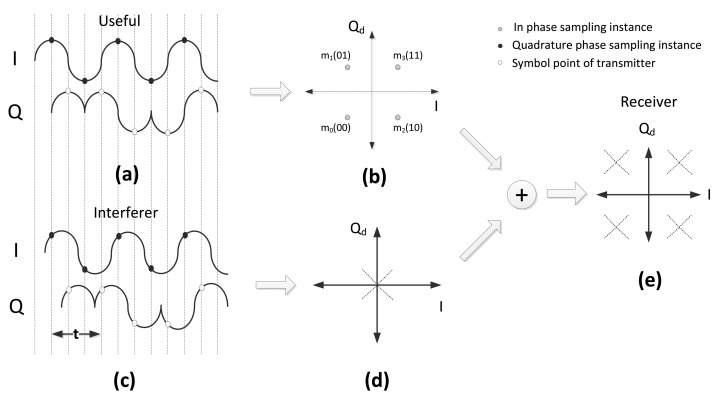
The impact of of the interferer: the asynchronous case. (**a**) Useful signal; (**b**) Q*_d_*-I domain representation of useful signal; (c) Interferer signal; (d) Q*_d_*-I domain representation of interferer signal; (e) Q*_d_*-I domain representation of received signal.

When there is perfect knowledge of the useful carrier phase and symbol timing, there is no difference, from the demodulator point of view, between half-sine-shaped or not shaped useful symbols, since the sampling is done at the maximum amplitude instance. As a consequence, the first addends in Equation (10) and Equation (12) are equal; this can also be observed by comparing Figure 1b with Figure 3b. When the pulse shaping function, $\cos(\frac{\pi t}{2T_{c}}+\phi_{I})$, of the interferer is included to the scenario, the interferer signal will be positioned on one of the diagonal lines shown in Figure 3d. As a consequence, the received signal plus interferer will be positioned over the dashed lines of Figure 3e. To obtain the error probability, we need to derive the probability distribution of the amplitude of the interferer at the sampling instance.

The random symbol sampling instance, *t*, and carrier phase, *ϕ_I_*, of the interferer can be modeled as uniformly distributed random variables. To simplify the notation in a pulse shaping function, we substitute the random variables as $X=\frac{\pi t}{2T_{c}}$, *Y* = *ϕ_I_* and *Z* = *X* + *Y* and denote the Probability Density Functions (PDFs) of *X* and *Y* as *f_X_*(*x*) and *f_Y_*(*y*); then the PDF of Z, which is our interest, will be the convolution integral [27] given as:

(13) $$\begin{array}{ll} f_{Z}(z)=\int_{-\infty }^{\infty }f_{X}(x)f_{Y}(z-x)dx & =\{\begin{array}{ll} \frac{\pi +2z}{4\pi^{2}} & \frac{-\pi }{2}\leq z<\frac{\pi }{2}\\ \frac{1}{2\pi } & \frac{\pi }{2}\leq z<\frac{3\pi }{2}\\ \frac{5\pi -2z}{4\pi^{2}}, & \frac{3\pi }{2}\leq z<\frac{5\pi }{2}\\ 0 & \text{otherwise} \end{array}\\ & \begin{array}{cc} =\frac{1}{2\pi } & 0\leq z<2\pi \end{array} \end{array}$$

where:

$$\begin{array}{cc} f_{X}(x)=\{\begin{array}{ll} \frac{1}{\pi }, & \frac{-\pi }{2}\leq x<\frac{\pi }{2}\\ 0 & \text{otherwise} \end{array} & f_{Y}(y)=\{\begin{array}{ll} \frac{1}{2\pi }, & 0\leq y<2\pi \\ 0 & \text{otherwise} \end{array} \end{array}$$

The solution of the convolution integral in Equation (13) indicates that *Z* is a uniformly-distributed random variable in the interval 0 ≤ *z* < 2*π*. Thus, the sampled point of the interferer signal is $\left(\pm \sqrt{\frac{I}{2}}\pm j\sqrt{\frac{I}{2}})\cos(z\right)$, and it always stays over the dashed lines in Figure 3d. If the interferer is transmitting *m*_3_ or *m*_0_, the slope of the diagonal line is +1; on the other hand, if the interferer is transmitting *m*_1_ or *m*_2_ then the slope is − 1. The probability distribution of the positions of the interferer sampling point on the dashed lines are symmetric on the opposite sides of the origin, because of the cosine function. The amplitude in a quadrant is:

(14) $$\begin{array}{cc} i=\sqrt{I}\cos(z) & -\pi /2\leq z<\pi /2 \end{array}$$

Using Equation (14), the PDF of the amplitude is written as:

(15) $$\begin{array}{cc} f_{i}(i)=\frac{2}{\pi \sqrt{1-{\left(i/\sqrt{I}\right)}^{2}}} & 0\leq i\leq \sqrt{I} \end{array}.$$

Then, we can also obtain the probability that the interferer amplitude lies in an interval (*a, b*] using Equation (15):

(16) $$\text{Prob}(a<i\leq b)=F_{i}(b)-F_{i}(a)=\int_{\frac{a}{\sqrt{I}}}^{\frac{b}{\sqrt{I}}}f_{i}(i)di=\frac{2}{\pi }(\sin^{-1}(\frac{b}{\sqrt{I}})-\sin^{-1}(\frac{a}{\sqrt{I}}))$$

where *F_i_*(*i*) is the Cumulative Distribution Function (CDF) of *i*.

Without loss of generality, as we did in Section 3.1, it can be assumed that the transmitter is transmitting m_3_. Therefore, to obtain *P_c_* and *P_s_*, we should consider the following two cases: (a) $\sqrt{I}<\sqrt{C}$, and (b) $\sqrt{I}\geq \sqrt{C}$

#### 3.2.1. Case (a): $\sqrt{I}<\sqrt{C}$

When $\sqrt{I}$ is smaller than $\sqrt{C}$, the demodulator never makes an error, since the received signal never crosses the other quadrants. Thus, *P_c_* and *P_s_* are equal to zero:

$$\begin{array}{ll} P_{s} & =0\\ P_{c} & =0 \end{array}$$

#### 3.2.2. Case (b): $\sqrt{I}\geq \sqrt{C}$

When *i*, which refers to the amplitude of the interferer signal at the sampling instance, is larger than or equal to $\sqrt{C/2}$, the receiver can decide for the wrong symbol with a probability of $\frac{3}{4}$. Regarding *P_c_*, a transition towards *m*_2_ or *m*_1_ will result in one chip error per symbol, while a transition towards *m*_0_ will result in two chip errors per symbol. Therefore, the error rates when $i>\sqrt{C/2}$ are:

$$\begin{array}{ll} P_{s} & =\frac{3}{4}\\ P_{c} & =\frac{1}{4}\cdot \frac{1}{2}+\frac{1}{4}\cdot \frac{1}{2}+\frac{1}{4}=\frac{1}{2} \end{array}$$

In general, *P_s_* and *P_c_* can be written as a function of *i* as follows:

$$\begin{array}{ll} P_{s}(i) & =\{\begin{array}{ll} 0, & i<\sqrt{C}\\ \frac{3}{4}, & \sqrt{C}\leq i\leq \sqrt{I} \end{array}\\ P_{c}(i) & =\{\begin{array}{ll} 0, & i<\sqrt{C}\\ \frac{1}{2}, & \sqrt{C}\leq i\leq \sqrt{I} \end{array} \end{array}$$

Finally, we can derive the error probabilities when $\sqrt{I}$ is greater than $\sqrt{C}$ by evaluating the expected values of *P_s_*(*i*) and *P_c_*(*i*):

$$\begin{array}{ll} P_{s} & =E[P_{s}(i)]=\int_{\sqrt{C}}^{\sqrt{I}}\frac{3}{4}f_{i}(i)di=\frac{3}{4}\cdot F_{i}(\sqrt{C}<i\leq \sqrt{I})=\frac{3}{4}\frac{2}{\pi }(\frac{\pi }{2}-\sin^{-1}(\sqrt{\frac{C}{I}}))=\frac{3}{4}-\frac{6}{4\pi }\sin^{-1}(\sqrt{\frac{C}{I}})\\ P_{c} & =E[P_{c}(i)]=\int_{\sqrt{C}}^{\sqrt{I}}\frac{1}{2}f(i)di=\frac{1}{2}\cdot F_{i}(\sqrt{C}<i\leq \sqrt{I})=\frac{1}{2}\frac{2}{\pi }(\frac{\pi }{2}-\sin^{-1}(\sqrt{\frac{C}{I}}))=\frac{1}{2}-\frac{1}{\pi }\sin^{-1}(\sqrt{\frac{C}{I}}) \end{array}$$

where *f_i_*(*i*) is given in Equation (15) and *F_i_*(*i*) is given in Equation (16).

The computational complexity of *P_s_* and *P_c_* are *O*(1) in big *O* notation, since *C* and *I* are constant values.

### 3.3. Validation of the Analytical Model through Simulations

**Figure 4.**
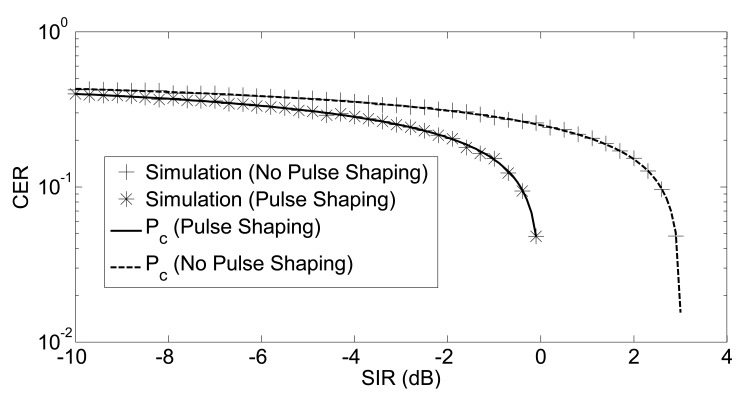
Chip error rate: the case of coherent O-QPSK demodulation. CER, chip error rate; SIR, signal-to-interference ratio.

The validity of our analytical framework has been tested using Monte-Carlo simulations. The simulation tool has been implemented in MATLAB (The MathWorks Inc., Natick, MA, USA) using the complex envelopes of the signals. Asynchronous symbols are obtained by shifting the complex envelope in the time axis, while the random carrier phase is obtained through rotating the complex envelope on the complex plane. SIR values with a step of 0.3 dB from −10 dB to 5 dB are simulated. At each specific SIR value, 10, 000 random O-QPSK symbols are generated for both the interferer and useful transmitter. The complex baseband representation of the O-QPSK symbol is generated by using 100 points; therefore, the resolution of the time for the asynchronous O-QPSK symbols was 10 ns. On the other hand, the resolution for the carrier phase was 2*π*/100. Random asynchronous interfering symbols with a random carrier phase are generated with uniformly-distributed parameters. The excellent agreement between simulation points and analytical curves is shown in Figure 4. Since the analysis in Sections 3.1 and 3.2 is exact (no approximation is used), we expect a perfect overlapping between simulations and the analytical model. Asymptotically, there is a 3-dB difference between the results of half-sine shaped and non-shaped symbols. In half-sine pulse shaping, *P_c_* goes to zero at 0 dB, while the threshold is 3 dB in the absence of pulse shaping.

## 4. Probability of Chip Error in Non-Coherent O-QPSK

**Figure 5.**
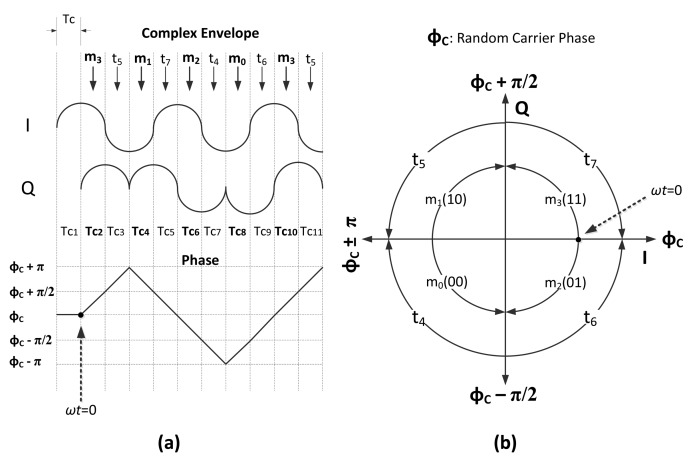
Phase transitions of the complex envelope. (**a**) Example phase transition sequence; (**b**) All possible phase transitions.

Normally, O-QPSK requires coherent detection [26]; however with the half-sine pulse shaping in 2.4 GHz PHY of IEEE 802.15.4, the information-bearing part of the signal is not only the carrier phase, but also the complex-envelope of the signal. Thus, without recovering the carrier phase, just observing the phase changes of the complex envelope, transmitted information can be extracted. In 2.4 GHz PHY of IEEE 802.15.4, the phase of the complex envelope increases or decreases by an amount of *π*/2 every *T_c_* seconds. In this sense, the behavior of the modulation is similar to that of minimum shift keying (MSK). The main difference between the two modulations is that they use different symbol mappings. In particular, symbol mapping in IEEE 802.15.4 is shown in Figure 5.

Chip intervals in Figure 5a are marked as *T_C_*_1_,*T_C_*_2_, …. In each even chip interval, *T_C_*_2_,*T_C_*_4_,…, a symbol (*m*_0_, *m*_1_, *m*_2_, *m*_3_) is transmitted, and in the odd chip intervals, *T_C_*_3_,*T_C5_*, …, there are phase changes (*t*_4_, *t*_5_, *t*_6_, *t*_7_), which can be considered as the transitions between two sequential O-QPSK symbols. For instance, in Figure 5a, a sequence of phase changes are shown. At the beginning of the *T_C_*_2_ chip interval (*i.e., ωt* = 0), the phase of the signal is equal to the carrier phase, *ϕ_C_*; then at the end of the same chip interval, the phase reaches *ϕ_C_* + *π*/2, which is indicated in Figure 5b by the arc, *m*_3_. In the next chip interval, *T_C_*_3_, the phase reaches the *ϕ_C_* + *π* value, which is represented by the arc, *t*_5_. The sequential chip intervals send the phase of the complex envelope, *π*/2, further or back with respect to the transmitted symbols. All possible phase transitions are shown in Figure 5b. This relationship between phase transition and transmitted symbol suggests the idea of constructing a non-coherent detector, which identifies the transmitted chip based on the phase transitions of the complex envelope. Using such a detector, *P_c_* will refer to the probability of the erroneous detection of phase transitions during a chip interval. In the absence of interference, the complex envelope of the received chip through a chip duration in the Q-I plane can be expressed as:

(17) $$\begin{array}{ccc} R(\omega t)=\sqrt{C}e^{j(\pm \omega t+\phi_{C})}, & \omega t∊[0,\pi /2], & \phi_{c}∊[0,2\pi ] \end{array}$$

where *C* is the energy of the signal and *ϕ_C_* is the uniformly distributed carrier phase. The definition domain of *ωt* is from zero to *π*/2, because the signal can only move *π*/2 further on the Q-I plane during a chip interval. The instantaneous phase of *R*(*ωt*) in Equation (17) is:

(18) $$\Theta_{R}(\omega t)=\pm \omega t+\phi_{C}$$

and the phase of the received signal at the beginning and at the end of the chip interval is:

$$\begin{array}{ll} \Theta_{R}(0) & =\phi_{C}\\ \Theta_{R}(\pi /2) & =\pm \pi /2+\phi_{C}. \end{array}$$

Since the carrier phase can be eliminated by subtracting Θ*_R_*(*π*/2) and Θ*_R_*(0), the following discriminator demodulator can be applied [28]:

(19) $$\Theta_{R}(\pi /2)\overset{+j}{\underset{-j}{≷}}\Theta_{R}(0)$$

where the decision is based on the comparison of the phases of the complex envelope of the received signal at the beginning and at the end of the chip interval. The demodulator decides for a positive phase change, +*j*, if the phase at the end of the chip interval is higher than the beginning; otherwise, it decides for a negative phase change, −*j*. Firstly, we will analyze the case where the symbols of the interferer are synchronous (according to the chip interval) with the useful symbols. This assumption is not realistic, but provides the mathematical basis for the framework that will be used for the more realistic case, where the interferer and useful symbols are asynchronous.

### 4.1. Interferer and Useful Symbols are Synchronous

When useful and interfering signals are synchronous, the received signal can be written as:

(20) $$R(\omega t)=\sqrt{C}e^{j(b_{C}\omega t+\phi_{C})}+\sqrt{I}e^{j(b_{I}\omega t+\phi_{I})}$$

where *ωt* = [0, *π*/2], *ϕ_C_* = [0, 2*π*] and *ϕ_I_* = [0, 2*π*]. *b_C_* and *b_I_* represent the turning directions of the phase of the complex envelope of the useful and the interferer chips on the complex domain in Figure 5b: *bC, bI* = +1 means a clockwise direction, whereas *b_C_, b_I_* = −1 means the counter-clockwise direction. We denote *P_eq_* as the probability of erroneous detection when the phases of useful and interferer signals are turning to the same direction (*b_C_* = *b_I_*), while *P_dif_* denotes the probability of erroneous detection when phase transitions are toward the different directions (*b_C_* ≠ *b_I_*). There are two cases; accordingly, *P_c_* is:

(21) $$P_{c}=\frac{1}{2}P_{eq}+\frac{1}{2}P_{dif}$$

Note that if the phase of the interferer signal turns toward the direction of the useful signal, the received signal in the Q-I plane will turn toward the direction of the useful signal, as well. As a consequence, *P_eq_* will always be zero. To obtain *P_dif_*, we can consider the case where *b_C_* = +1 and *b_I_* = −1; in fact, this is mathematically identical to the case of *b_C_* = −1 and *b_I_* = +1. Motivated by this consideration, we can define the following success/error rule for the demodulator, based on the phase values at the beginning and at the end of the chip interval:

(22) $$\Theta_{R}(\pi /2)\overset{s}{\underset{e}{≷}}\Theta_{R}(0)$$

where *s* means success and *e* means error. When *b_C_* = +1 and *b_I_* = −1, the received signal is:

(23) $$R(\omega t)=\sqrt{C}e^{j(\omega t+\phi_{C})}+\sqrt{I}e^{j(-\omega t+\phi_{I})}$$

To obtain an analytical expression to Θ*_R_*(*ωt*), we assume that during a chip interval, *R*(*ωt*) always stays on the definition domain of arctan (*i.e.*, [−*π*/2 *π*/2]). Starting from Equation (23), the instantaneous phase function can be written as:

(24) $$\Theta_{R}(\omega t)=\arctan\left(\frac{\sqrt{C}\sin(\omega t+\phi_{C})+\sqrt{I}\sin(-\omega t+\phi_{I})}{\sqrt{C}\cos(\omega t+\phi_{C})+\sqrt{I}\cos(-\omega t+\phi_{I})}\right)$$

With no loss of generality, we can assume *ϕ_c_* = 0. Now, after some trigonometric simplifications, the phases of the received signal at the beginning and at the end of the chip interval can be written as:

$$\begin{array}{ll} \Theta_{R}(0) & =\arctan\left(\frac{\sqrt{I}\sin(\phi_{I})}{\sqrt{C}+\sqrt{I}\cos(\phi_{I})}\right)\\ \Theta_{R}(\pi /2) & =\arctan\left(\frac{\sqrt{C}-\sqrt{(}I)\cos(\phi_{I})}{\sqrt{I}\sin(\phi_{I})}\right) \end{array}$$

After replacing Θ*_R_*(0) and Θ*_R_*(*π*/2) in Equation (22), the decision rule becomes:

(25) $$\arctan\left(\frac{\sqrt{C}-\sqrt{I}\cos(\phi_{I})}{\sqrt{I}\sin(\phi_{I})}\right)\overset{s}{\underset{e}{≷}}\arctan\left(\frac{\sqrt{I}\sin(\phi_{I})}{\sqrt{C}+\sqrt{I}\cos(\phi_{I})}\right)$$

by recalling that arctan(·) is monotonically increasing in [−*π*/1 *π*/2], Equation (25) becomes:

(26) $$\frac{\sqrt{C}-\sqrt{I}\cos(\phi_{I})}{\sqrt{I}\sin(\phi_{I})}\overset{s}{\underset{e}{≷}}\frac{\sqrt{I}\sin(\phi_{I})}{\sqrt{C}+\sqrt{I}\cos(\phi_{I})}$$

to keep the trajectory of *R*(*ωt*) in the definition domain of arctan, we can restrict the definition domain of *ϕ_I_* as [0,*π*/2]. With this assumption, $\sqrt{C}+\sqrt{I}\cos(\phi_{I})$ and $\sqrt{I}\sin(\phi_{I})$ will be always positive. After some algebra, Equation (26) becomes:

$$C-I\cos^{2}(\phi_{I})\overset{s}{\underset{e}{≷}}I\sin^{2}(\phi_{I})$$

which gives:

(27) $$C\overset{s}{\underset{e}{≷}}I$$

It can be showed that (27) holds for the other possible domain definitions of *R*(*ωt*); therefore, *P_dif_* is:

(28) $$\begin{array}{cc} P_{dif}=1 & C<I \end{array}$$

(29) $$\begin{array}{cc} P_{dif}=0 & C>I \end{array}$$

By replacing Equation (28) in Equation (21), the chip error probability becomes:

$$\begin{array}{lll} P_{c} & =1/2 & C<I\\ P_{c} & =0 & C>I \end{array}$$

### 4.2. Useful and Interfering Symbols are Asynchronous

The condition in which interfering symbols can be asynchronous with respect to useful symbols increases the number of possible cases to be considered for the evaluation of Θ*_R_*(*ωt*). In particular, the interferer signal can change its turning direction at a random instance, *t_t_*, and the corresponding random angle is *ϕ_t_* = *ωt_t_*. Consequently, the received signal during a chip interval can be written as:

(30) $$R(\omega t)=\{\begin{array}{ll} \sqrt{C}e^{j(b_{C}\omega t+\phi_{C})}+\sqrt{I}e^{j(b_{I_{1}}\omega t+\phi_{I})} & \omega t=[0,\phi_{t}]\\ \sqrt{C}e^{j(b_{C}\omega t+\phi_{C})}+\sqrt{I}e^{j(b_{I_{2}}\omega t+d)} & \omega t=(\phi_{t},\pi /2] \end{array}$$

where *b_C_* defines the turning direction of the useful chip, while *b*_*I*_1__ and *b*_*I*_2__ are the turning directions of the sequential chips of the interferer. In Equation (30), at the angle *ϕ_t_*, the interferer starts transmitting another chip. We denote this asynchronous interferer case probability as *P_mix_*. The occurrences of *P_eq_, P_dif_* (which are given in Section 4.1) and *P_mix_* for the different combinations of *b_C_*, *b*_*I*_1__ and *b*_*I*_2__ are shown in Table 2.

By considering these occurrences, the chip error probability can be evaluated as:

(31) $$P_{c}=1/4P_{eq}+1/4P_{dif}+1/2P_{mix}$$

To obtain *P_mix_*, we consider the case of *b_C_* = +1, *b_I_*_1_ = +1, *b_I_*_2_ = −1, and *d* = *ϕ_I_* + 2*ϕ_t_*, which is mathematically identical to the all other *P_mix_* cases in Table 2. Following the same approach followed in Section 4.1, we can write the decision rule as:

(32) $$\frac{\sqrt{C}-\sqrt{I}\cos(\phi_{I}+2\phi_{t})}{\sqrt{I}\sin(\phi_{I}+2\phi_{t})}\overset{s}{\underset{e}{≷}}\frac{\sqrt{I}\sin(\phi_{I})}{\sqrt{C}+\sqrt{I}\cos(\phi_{I})}$$

which can be simplified to:

(33) $$C+\sqrt{CI}(\cos(\phi_{I})-\cos(\phi_{I}+2\phi_{t}))-I\cos(2\phi_{t})\overset{s/e}{\underset{e/s}{≷}}0$$

where the sign in the inequality depends on the sign of denominators in Equation (32). Equation (33) can be rearranged as:

(34) $$C/I+\sqrt{C/I}(\cos(\phi_{I})-\cos(\phi_{I}+2\phi_{t}))-\cos(2\phi_{t})\overset{s/e}{\underset{e/s}{≷}}0$$

*P_mix_* is obtained by using the test in Equation (34) considering the different signs of the denominators in Equation (32) and the definition domain of the arctan function.

### 4.3. Validation of the Analytical Model through Simulations

**Figure 6.**
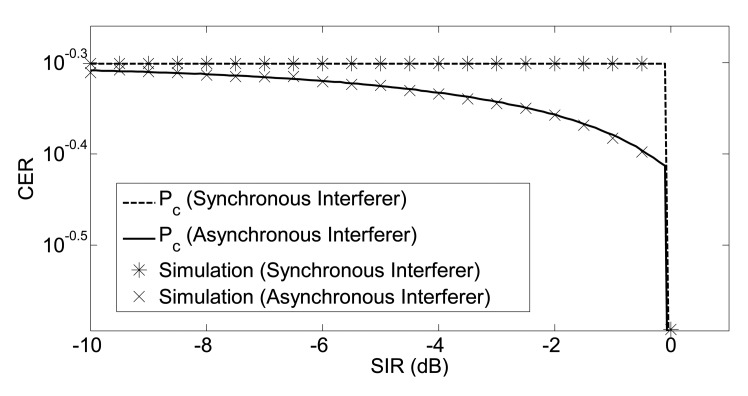
Non-coherent chip error rate.

Simulation results for the cases of synchronous and asynchronous useful and interfering symbols are shown in Figure 6. Since the analysis in Section 4 is exact, we expect a perfect agreement between simulations and analytical model. The results of Figure 6 confirm this intuition.

## 5. Probability of Data Error in IEEE 802.15.4

The received path of the commercial 802.15.4 compatible transceivers, such as TICC2420 [29] and Freescale MC13224 [30], is composed of two main blocks: an O-QPSK demodulator and a de-spreader to obtain the data symbols from the received chips. In the previous section, we have obtained the chip error rate, *P_c_*, by assuming both coherent and non-coherent detection; this error probability is used here to derive bounds for the symbol error probability, *P_d_*, and for the corresponding packet reception rate, *P_r_*.

### 5.1. Chip Error Rate to Data Symbol Error Rate

As discussed previously, in the 2.4 GHz PHY of IEEE 802.15.4, data symbols are mapped to the chips after the demodulation. Because of the symmetry in the data symbol set used by the 802.15.4 2.4 GHz PHY layer, the error probability of the data symbol, *S*_0_, is equal to the symbol error probability, *P_d_* [31]:

(35) $$\begin{array}{cc} P_{d}=P(e|S_{0})=P(e|S_{i}) & \text{for}\;i=1,\ldots ,15 \end{array}$$

Hence, with no loss of generality to obtain an analytical expression for *P_d_*, we can find the union upper bound of *P*(*e*|*S*_0_) expressed as:

(36) $$P(e|S_{0})\leq \overset{15}{\underset{i=1}{\text{∑}}}P(S_{i}|S_{0})$$

where *P*(*S_i_*|*S*_0_) represents the probability of deciding for the symbol, *S_i_*, once symbol *S*_0_ has been transmitted. Each probability in the summation of Equation (36) can be formulated as a function of Hamming distance, *h_i_*, between *S*_0_ and *S_i_*, and the chip error rate, *P_c_*, as:

(37) $$P(S_{i}|S_{0})=P(h_{i},P_{c})=\{\begin{array}{ll} \frac{1}{2}\left(\begin{array}{c} h_{i}\\ h_{i}/2 \end{array}\right)P_{c}^{h_{i}/2}{\left(1-P_{c}\right)}^{h_{i}/2}+{\text{∑}}_{i=\frac{h_{i}+2}{2}}^{h_{i}}\left(\begin{array}{c} h_{i}\\ i \end{array}\right)P_{c}^{i}{\left(1-P_{c}\right)}^{h_{i}/i} & \text{if}\;h_{i}\;\text{is even}\\ {\text{∑}}_{i=\frac{h_{i}+1}{2}}^{h_{i}}\left(\begin{array}{c} h_{i}\\ i \end{array}\right)P_{c}^{i}{\left(1-P_{c}\right)}^{h_{i}-i} & \text{if}\;h_{i}\;\text{is odd} \end{array}$$

By substituting Equation (37) in Equation (36), we obtain an upper bound that can be applied to any symmetric symbol set. The bound will be used in the next sub-sections to obtain expressions for the data symbol error probability of both coherent and non-coherent detection.

#### 5.1.1. Coherent Detection

In the case of coherent detection, we can calculate the Hamming distance of each chip sequence. In particular, the recurrences of the Hamming distances of the data symbols is given in Table 3.

Using these recurrences, the symbol error probability can be bounded as follows:

(38) $$P_{d}\leq \overset{N}{\underset{i=1}{\text{∑}}}r_{i}P(h_{i},P_{c})=2P(12,P_{c})+2P(14,P_{c})+3P(16,P_{c})+2P(18,P_{c})+6P(20,P_{c})$$

where *P*(*h_i_, P_c_*) is taken from Equation (37) and *h_i_* and *r_i_* are given in Table 3. *i* is the index for columns, and *N* = 5 is the number of columns in the table.

Note that, for a fixed value of *P_c_*, the computational complexity of the previous upper bound is *O*(1).

#### 5.1.2. Non-Coherent Detection

In the non-coherent case, we can only identify the phase transitions during the chip intervals. For a 32-chip long sequence, there will be 31 phase transitions, as shown in Table 4.

Where + means a *π*/2 increase in the phase and − means a *π*/2 decrease in the phase. We can think of the phase transition sequence of a data symbol in Table 4 as a vector in Hamming space and calculate the Hamming distances between these vectors. The recurrences of Hamming distances from a data symbol to the other data symbols in the set are given in Table 5.

The union upper bound is obtained as:

(39) $$P_{d}\leq \overset{N}{\underset{i=1}{\text{∑}}}r_{i}P(h_{i},P_{c})=2P(13,P_{c})+2P(14,P_{c})+3P(15,P_{c})+3P(16,P_{c})+2P(17,P_{c})+2P(18,P_{c})+P(31,P_{c})$$

where *h_i_* and *r_i_* are given in Table 5. *i* is the index for columns, and *N* = 7 is the number of columns in the table.

Again, the computational complexity of the upper bound is *O*(1).

### 5.2. Packet Reception Rate

In 802.15.4 2.4 GHz PHY, four data bits are mapped to a data symbol [2]. When a packet of *b*-bytes is transmitted, the probability of successfully receiving the packet, *P_r_*, is calculated as:

(40) $$P_{r}={\left(1-P_{d}\right)}^{2b}$$

**Figure 7.**
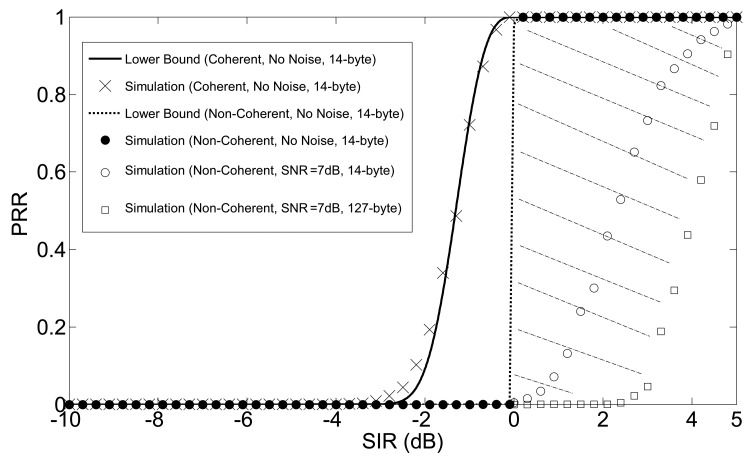
Packet reception rate (PRR).

It is worth noting that the upper bounds on *P_d_* in Equation (38) and Equation (39) will become lower bounds in Equation (40). *P_r_* as a function of SIR is shown in Figure 7 for the coherent and non-coherent demodulation schemes considered in Sections 3 and 4. Comparison between analysis and simulations is also shown. Results for the case of no thermal noise (cross-points for coherent and filled circles for non-coherent) have been obtained for a PHY service data unit (PSDU) of 14 bytes. The figure shows that the agreement between analysis and simulations is very good.

Experimental results shown in Figure 7 of [19] suggest 7 dB for the threshold of SNR, where the performance of the system, in terms of PRR, completely switches from zero to one. In this sense, the interval from *SNR* = 7 dB to *SNR* = ∞ should be adequate for representing a great variety of different SNR operation conditions, where the noise is not dominant, but the performance of the system is mostly determined by the interferer. This interval results in a considerably narrow region of PRR (the shaded area in Figure 7), and the experiment results in [16,18–20] fall into this region; therefore, it would not be wrong to conclude that the mathematical analysis presented here captures the essential features of the real-world performance, where the dominant factor of performance degradation is interference. Two different values of PSDU are considered in Figure 7: 14 bytes (experiments in [20]) and 127 bytes (the maximum allowable, which is used in experiments in [16,18,19]). Finally, It is also worth noting that 0 dB is the absolute minimum SIR necessary to successfully detect the useful packets in non-coherent demodulation.

## 6. Conclusion

The effect of concurrent transmission on the performance, in terms of chip, symbol and packet error probability, of IEEE 802.15.4 systems was investigated. To this aim, an analytical model, which starts from the PHY characterization of the IEEE 802.15.4 standard, was proposed, and the results were validated using simulations. Since the system performance does not depend on the number of interfering devices, but rather, on the overall amount of interfering power [18,20], the case of one single interferer was considered. Both non-coherent and coherent demodulation were analyzed in the absence of thermal noise. The joint effect of thermal noise and interference was taken into account by means of simulations. For the case of non-coherent demodulation, we found that 0 dB is the absolute minimum necessary value of SIR for the successful detection of the useful packets. We have also shown that the proposed analysis is in agreement with the measurement results of the literature [16,18–20] under realistic working conditions. We conclude that the mathematical framework can be used to provide analytical explanations to the experimental results shown in [16,18–20].

## Figures and Tables

**Table 1.** Signal reception models in network simulators [24]. SNRT, signal-to-noise ratio threshold; BER, bit error rate.

**Simulator**	GloMoSim	ns-2	OPNET
**Signal Reception**	SNRT-based, BER-based	SNRT-based	BER-based

**Table 2.** Useful and Interferer Chip Phase Combinations.

***b****_C_*	***b****_I_***_1_**	***b****_I_***_2_**	**d**	**Probability of Error**
+1	+1	+1	*ϕ_I_*	*P_eq_* = 0
+1	+1	−1	*ϕ_I_* + 2*ϕ_t_*	*P_mix_*
+1	−1	+1	*ϕ_I_* − 2*ϕ_t_*	*P_mix_*
+1	−1	−1	*ϕ_I_*	*P_dif_*
−1	+1	+1	*ϕ_I_*	*P_dif_*
−1	+1	−1	*ϕ_I_* + 2*ϕ_t_*	*P_mix_*
−1	−1	+1	*ϕ_I_* − 2*ϕ_t_*	*P_mix_*
−1	−1	−1	*ϕ_I_*	*P_eq_* = 0

**Table 3.** Recurrences of unique Hamming distance values.

*H_I_*	12	14	16	18	20
Reoccurrence (*R_I_*)	2	2	3	2	6

**Table 4.** Phase transitions of chip sequences in 2450-MHz PHY of IEEE 802.15.4.

**Data Symbol**	**Data Bits**	**Phase Transitions**
*S*_0_	0000	++------+++-++++-+-+++--++-++--
*S*_1_	1000	+--+++------+++-++++-+-+++--++-
*S*_2_	0100	++-++--+++------+++-++++-+-+++-
*S*_3_	1100	++--++-++--+++------+++-++++-+-
*S*_4_	0010	-+-+++--++-++--+++------+++-+++
*S*_5_	1010	++++-+-+++--++-++--+++------+++
*S*_6_	0110	+++-++++-+-+++--++-++--+++-----
*S*_7_	1110	----+++-++++-+-+++--++-++--+++-
*S*_8_	0001	--++++++---+----+-+---++--+--++
*S*_9_	1001	-++---++++++---+----+-+---++--+
*S*_10_	0101	--+--++---++++++---+----+-+---+
*S*_11_	1101	--++--+--++---++++++---+----+-+
*S*_12_	0011	+-+---++--+--++---++++++---+---
*S*_13_	1011	----+-+---++--+--++---++++++---
*S*_14_	0111	---+----+-+---++--+--++---+++++
*S*_15_	1111	++++---+----+-+---++--+--++---+

**Table 5.** Recurrences of unique Hamming distance values.

*h_i_*	13	14	15	16	17	18	31
**reoccurrence** (*r_i_*)	2	2	3	3	2	2	1
